# Relationship of Main Pulmonary Artery (Truncus Pulmonalis) Diameter With Hospital Stay and Mortality in Pulmonary Hypertension Patients Admitted to the Emergency Department

**DOI:** 10.7759/cureus.47918

**Published:** 2023-10-29

**Authors:** Mehmet Cagrı Goktekin, Feyza Aksu, Ahmet Zafer Perilioglu, Ramazan Fazil Akkoc

**Affiliations:** 1 Department of Emergency Medicine, Faculty of Medicine, Firat University, Elazığ, TUR; 2 Department of Anatomy, Faculty of Medicine, Firat University, Elazığ, TUR

**Keywords:** large blood vessels, mortality, emergency department, pulmonary hypertension, main pulmonary artery (truncus pulmonalis) diameter

## Abstract

Introduction: Pulmonary hypertension (PH) is a haemodynamic and pathophysiological disease significantly associated with morbidity and mortality. The increase in pulmonary vascular resistance, high pulmonary artery pressure and wall tension that occurs in PH results in dilatation of the main pulmonary artery (truncus pulmonalis), one of the largest and most important vessels in the body. The aim of this study is to investigate the relationship between the diameter of the truncus pulmonalis and hospitalization, length of hospital stay, and mortality in patients diagnosed with PH.

Methods: Demographic characteristics, number of Emergency Department (ED) admissions, post-admission status, treatment, truncus pulmonalis diameter, and mortality were evaluated statistically through the patient files of 115 PH patients who presented to the ED of Fırat University Faculty of Medicine, Elazığ, Türkiye, between January 2022 and December 2022.

Results: Of the 115 PH patients who came to the ED, 70 (60.8%) were women and 45 (39.2%) were men, with a mean age of 78.77±8.72 years. Fifty-one of these patients were discharged from the ED after treatment, and 64 were hospitalized. The mean length of hospital stay was two (min=0, max=38) days. Thoracic CT scans demonstrated that the mean diameter of the truncus pulmonalis of the patients was 34.874±3.288 mm (35.20±3.6509 mm in women, 34.367±2.5836 mm in men; p₌0.351) and there was no statistically significant relationship with mortality (p=0.496), hospitalization (p=0.806), and length of hospital stay (p=0.416). There was a statistically significant relationship between mortality rate and male gender (p=0.02) and comorbidity (p=0.001).

Conclusion: It was determined that there was no statistically significant relationship between the truncus pulmonalis diameter and gender, comorbidity, hospitalization, length of hospital stay, and mortality in this study in which single-centre one-year admissions of PH, which differ in aetiology, epidemiology, and demographic features were examined. However, among the patient demographics, a significant relationship was determined between gender and the number of comorbidities and mortality.

## Introduction

Pulmonary hypertension (PH) is a hemodynamic and pathophysiological disease characterized by increased pulmonary vascular resistance and pulmonary artery pressure (PAP) and is significantly associated with morbidity and mortality. In PH, the increase in mean PAP determined by right heart catheterization is defined as ≥25 mmHg [1-3]. Increased pulmonary vascular resistance scales up higher PAP and wall tension, resulting in dilatation of the main pulmonary artery (truncus pulmonalis).

The main pulmonary artery is one of the body's largest and most important vessels. It carries blood from the right side of the heart to the lungs for oxygenation. It initially courses in front of the ascending aorta, and as it ascends, it courses posteriorly to the left of the ascending aorta and divides into the right and left pulmonary arteries at the level of the fifth thoracic vertebra (T5) [4]. Dilation of the truncus pulmonalis can be seen on plain radiography or echocardiography, but computed tomography (CT) or magnetic resonance imaging (MRI) of the thorax provides a more accurate measurement [5]. The main pulmonary artery is approximately 5 cm in length and 3 cm in diameter. In a study conducted by Karazincir et al. on healthy adults, they reported the mean main pulmonary artery diameter as 27.0 ± 2.8 mm in men and 25.9 ± 3.0 mm in women [6]. In the same study, it was reported that the diameter increased in line with age and body surface area.

The aetiology, epidemiology and demographic characteristics vary in PH disease. PH aetiology is evaluated in five groups according to clinical symptoms and pathological findings. Classical PH is classified as Group 1, PH due to left heart disease as Group 2, PH due to pulmonary disease or hypoxia as Group 3, PH due to chronic thromboembolic as Group 4, and multifactorial PH as Group 5 [7].

The epidemiology of PH, which affects 15-60 million people worldwide [8], varies depending on the clinical condition in which it occurs. The prevalence of PH was determined a 10.9% in a study conducted with 4579 patients [9]. Of these, 78.7% were determined to be due to left heart disease, 9.7% to lung disease, 4% to hypertension, 0.6% to chronic thromboembolic hypertension, and 6.8% to unidentified causes. Its prevalence is two to three times higher in women than in men [9].

PH displays a progressive clinical course. Exacerbations that lead to hospitalization are common in patients, which corresponds to 12.8% of patients presenting to the ED [10]. Examination of comorbidities shows that PH occurs in approximately 10% of patients with connective tissue disease and approximately half of these patients die within one year [11,12]. The mortality rate is reported to increase two to three times in PH patients occurring as a complication of idiopathic pulmonary fibrosis and two times in PH patients with sarcoidosis [13,14].

All patients diagnosed with PH, which causes significant use of resources [15,16], who came to a single ED, were evaluated in this study. Demographic characteristics of PH patients, the number of ED admissions, post-admission status, the main pulmonary artery diameter, and its relationship with hospitalization, length of hospital stay, and mortality were evaluated.

## Materials and methods

This retrospective study was initiated after the approval obtained from the Firat University Non-Interventional Research Ethics Committee (approval number: 2023/13-15). Patients diagnosed with PH who came to Fırat University Faculty of Medicine ED between January 2022 and December 2022 were retrospectively examined. Demographic characteristics of PH patients, number of ED admissions, post-admission status, treatment, diameter of the main pulmonary artery measured by imaging, accompanying comorbidities, and mortality were evaluated statistically.

**Figure 1:**
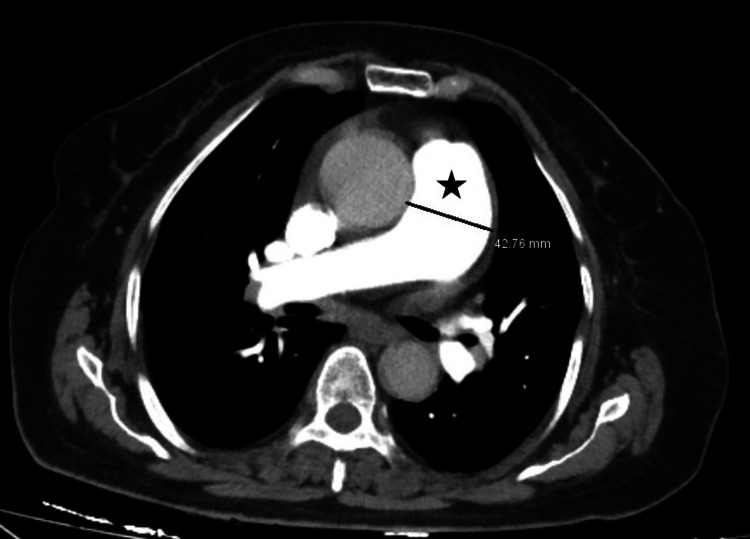
Axial CT measuring the diameter of the main pulmonary artery (marked by star) in mediastinal window setting

Reports of measurements made by two radiologists were used in the study. Contrast-enhanced CT images of the patients were used. The device used used in the shooting was the Revolution™ CT scanner (GE HealthCare Technologies, Inc., Chicago, Illinois, United States) with 128 detectors and 256 slices. The transverse axial section diameter of the main pulmonary artery was measured at the level of the bifurcation of the right pulmonary artery, in axial section in a mediastinal window setting (centre 40, width 400) (Figure 1).

Statistical analysis

IBM SPSS Statistics for Windows, Version 22.0 (Released 2013; IBM Corp., Armonk, New York, United States) was used to analyze the data. Descriptive statistics were presented as mean±SD, median (min-max) for quantitative data, and frequency and percentage for qualitative data. Conformity of the data to normal distribution was evaluated with the Kolmogorov-Smirnov test. For comparison of quantitative measurements between two independent groups, the Mann-Whitney U test was used for variables that did not conform to normal distribution. The Krusskal-Wallis-H test was used to compare quantitative measurements between more than two independent groups. The Pearson-Chi square test was used for categorical variables. The level of significance was accepted as 0.05.

## Results

In 2022, a total of 115 PH patients, 70 (60.8%) women and 45 (39.2%) men, presented at the ED of the Fırat University Faculty of Medicine Hospital. The mean age of the patients was determined as 78.77±8.72 years. Fifty-one of these patients were discharged from the ED after treatment and 64 were admitted to the hospital (Table 1). The mean length of hospitalization following admittance to the ED is two (min=0, max=38) days.

**Table 1:** Departments where pulmonary hypertension patients were hospitalized

Department	Number of patients (n)	Percentage (%)
Intensive Care Unit (ICU)	30	46.8
Chest Diseases	20	31.2
Coronary ICU	6	9.4
Infectious Diseases	3	4.7
Neurology	2	3.1
Internal Diseases	1	1.6
Hematology	1	1.6
Nephrology	1	1.6
Total	64	100

The treatment applied to PH patients included medicinal treatment(75.7%), invasive treatment (13.9%), and non-invasive treatment (10.4%). Information about the number of ED admissions in one year of the 115 PH patients is given in Table 2.

**Table 2:** Number of ED admissions of pulmonary hypertension patients

Number of ED admissions in one year	Number of patients (n)	Percentage (%)
1	70	60.8
2	14	12.2
3	14	12.2
4	4	3.5
5	5	4.3
6	3	2.6
7	2	1.7
8	1	0.9
9	1	0.9
13	1	0.9
Total Patients	115	100

Thoracic CT was the imaging method for PH patients who were admitted to the ED. The mean main pulmonary artery diameter of PH patients was 34.874±3.288 mm (35.20±3.6509 mm in women, 34.367±2.5836 mm in men, p=0.351). The relationship between the main pulmonary artery diameter measured on imaging and mortality (p=0.496), hospitalization (p=0.806), and length of hospital stay (p=0.416) was not determined to be statistically significant. Information about mortality and hospitalization is presented in Table 3 and Table 4.

**Table 3:** Relationship between main pulmonary artery diameter and mortality

Post-admission status	Number of patients (n)	Main pulmonary artery diameter (mm)
Healthily discharged	91	34.995±3.3545
Mortality	24	34.417±3.0455

**Table 4:** Relationship between main pulmonary artery diameter and hospitalization

Post-admission status	Number of patients (n)	Main pulmonary artery11 diameter (mm)
Discharged	51	34.990±3.6803
Hospitalized	64	34.781±2.9652

Within the one-year period, 24 of 115 patients (20.9%) who came to the ED died, 20 of whom were admitted to the ICU. Evaluation of mortality among PH patients according to gender showed a statistically significant difference (p=0.02), revealing that eight out of 70 female patients and 16 out of 45 male patients died.

Accompanying comorbidities of PH patients were classified according to the diseases involved in the aetiology. Groups were graded from having no comorbidities to having four different comorbidities (Group 1: no comorbidities, Group 2: one additional disease according to aetiology, Group 3: two additional diseases according to aetiology, Group 4: three additional diseases according to aetiology, Group 5: four additional diseases according to aetiology). The relationship between the number of comorbidities and main pulmonary artery diameter was not statistically significant (p=0.658). A significant relationship (p=0.001) was detected between the number of comorbidities and mortality (Table 5).

**Table 5:** The relationship between accompanying comorbidities and mortality

Comorbidity	Healthily discharged	Mortality	Total
Group 1	11 (100%)	0 (0%)	11 (100%)
Group 2	22 (78.6%)	6 (21.4%)	28 (100%)
Group 3	40 (81.6%)	9 (18.4%)	49 (100%)
Group 4	17 (89.5%)	2 (10.5%)	19 (100%)
Group 5	1 (12.5%)	7 (87.5%)	8 (100%)

Of the 115 PH patients, 13 (11.3%) had an accompanying rheumatological disease. Six such patients were discharged from the ED and seven were hospitalized; of the seven hospitalized patients, three died. Twenty-five PH patients had additional respiratory system-related diseases (e.g. asthma, chronic obstructive pulmonary disease (COPD), asbestos), of which eight (32%) were discharged and 17 (68%) were hospitalized. Five of these 25 patients (20%) died.

## Discussion

In this single-centre study, which included PH patients who presented to the ED, the mean age of the patients was approximately 78 years old and the majority were female. The patients had more than one comorbid disease with a rate as high as 75.6%. Consistent with literature studies, the majority of PH patients were accompanied by cardiovascular diseases, lung disease, or other secondary diseases [1,17].

Considering the number of admissions of PH patients who presented to the ED during the year, it was determined that 11.3% (average 6.6 applications) applied frequently. It was determined that the most frequent ED admission was of one patient with a total of 13 visits within the year. The majority of frequently admitted patients were female, and there was a high rate of concomitant hypertension and congestive heart failure. Although the frequency of admission is consistent with the literature, the demographic characteristics and comorbidities of the patients vary [17]. While more than half of the patients who frequently came to the ED were hospitalized, it was observed that the majority of these patients were admitted to the ICU.

It was determined that 55.6% of PH patients were hospitalized after presenting to the ED. The mortality rate in PH patients who came to the ED was 20.8%, which was determined to be higher in patients who were hospitalized (37.5%). The data obtained in this study are consistent with the literature [17,18].

In healthy adults, the main pulmonary artery diameter was approximately 30 mm and was bigger in men than in women. When compared to studies in the literature in which the main pulmonary artery diameter was measured in healthy adult individuals [6,19], an increase was observed in the diameter. ​In addition, Karazincir et al. reported that the upper threshold of the main pulmonary artery diameter in individuals with normal PAP was 32.6 mm [6]. In this study, the average main pulmonary artery diameter of all patients was found to be higher than 32.6 mm. While the main pulmonary artery diameter is larger in males than in females in the healthy population, it was determined that PH enlarged the diameter of the main pulmonary artery in females to a greater extent in the current study. This brought the following question to mind: “Could there be a relationship with higher mortality in men who display diameter enlargement at a lesser extent compared to women?”. However, the current study had a limited population, and more extensive studies with a greater population are needed to answer this question.

Many studies have reported that an increase in main pulmonary artery diameter increases mortality in some diseases (e.g., PH, coronavirus disease 2019 (COVID-19)) [18,20-22]. According to the data obtained in this study, it was determined that the increase in main pulmonary artery diameter was not significantly associated with mortality. However, mortality was statistically significantly higher in men than in women.

Evaluation of the comorbidities in patients diagnosed with PH shows that the most three common include hypertension, congestive heart failure, and diabetes mellitus. The results showed that the presence of additional respiratory system diseases in patients did not increase mortality, but increased the hospitalization percentage. Among comorbidities, it was determined that the presence of rheumatological disease affected mortality (23.07%).

The limitations of this study were its retrospective nature, low number of patients, and the single-centre setting.

## Conclusions

There are available studies on PH and its demographics in the literature. However, the relationship between main pulmonary artery diameter, mortality, number of comorbidities, and gender appears somewhat contradictory in other studies as compared to this study. There may be a relationship between gender-related main pulmonary artery diameter and mortality in PH patients. In this regard, we believe that studies with a greater number of patients and more data are needed to reduce mortality and morbidity in PH patients and to improve health services. 
